# Can social robots improve the hospital experience of children?

**DOI:** 10.3389/fresc.2026.1691526

**Published:** 2026-02-26

**Authors:** Jorge Buele, Christian Junta-Andagana, Marcelo Fajardo-Pruna, Edwin Pozo-Safla, Francisco Yumbla

**Affiliations:** 1 Centro de Investigación en Mecatrónica y Sistemas Interactivos (MIST), Facultad de Ingenierías, Universidad Tecnológica Indoamérica, Ambato, Ecuador; 2 Facultad de Ingeniería en Mecánica y Ciencias de la Producción, Escuela Superior Politécnica del Litoral (ESPOL), Guayaquil, Ecuador; 3 Facultad de Mecánica, Escuela Superior Politécnica de Chimborazo (ESPOCH), Riobamba, Ecuador

**Keywords:** assistive technologies, caregivers, child anxiety, pediatric hospitalization, social robots

## 1 Introduction

Child hospitalization constitutes a critical event that frequently disrupts the daily lives of children and their families (1). Beyond the biomedical dimension, hospital admission processes generate ruptures in routines, social bonds, and play experiences, which can increase the psychological and educational vulnerability of pediatric patients. This situation is linked to a broader challenge: how to ensure that assistive technologies also respond to the emotional needs that arise in hospital environments (2).

In this context, social robots have emerged as a visible and rapidly developing branch of assistive technology in pediatric settings. These systems are understood as physically embodied interactive technologies designed to engage users through social behaviors such as communication, emotional expression, or relational interaction, distinguishing them from purely digital applications or passive entertainment devices (3). In recent years, advances in social robotics and supportive technological environments have enabled the incorporation of tools that, without replacing healthcare personnel, complement care through interventions aimed at improving the hospital experience. As these technologies expand across clinical and research contexts, questions remain regarding their scope, intended use, and evaluation. Should they be conceived only as devices for entertainment or distraction, or can they be consolidated as instruments that strengthen children's emotional resilience and expand the care capacity of the hospital system?

As in other areas of disability and rehabilitation research, where the importance of combining objective and subjective measures to capture patients' lived experience is debated (4), the field of assistive technology in pediatric hospitalization faces the need to transcend the strictly technical paradigm. Rather than focusing exclusively on hardware performance or design characteristics, it becomes important to consider how these tools are positioned within clinical routines, how their effects are framed and assessed, and how they contribute to broader inclusion and well-being goals promoted by organizations such as the WHO.

This article seeks to contribute to that debate with a critical reflection grounded in recent literature and studies applied in hospital contexts. It aims to assess the methods used, identify strengths and weaknesses of current hypotheses, and open a constructive discussion on the relevance of social robots as assistive technology within the healthcare framework.

## 2 Clinical evidence in pediatric hospitalization

It is difficult to continue discussing the role of social robots in pediatrics as if they were merely “technological novelties.” Growing evidence indicates that these systems can support the clinical and emotional needs of hospitalized children when integrated into existing care pathways. Playful interaction with robots such as CoderBot (an interactive robot platform) (5), PLEO (a dinosaur-like robotic companion) (6), aibo (a pet-like social robot) (7) or projects such as the Baby Goldrake humanoid robot does not replace medical treatments. Rather, it can expand the hospital system's capacity to deliver more comprehensive care by addressing psychological well-being alongside physical health. This shift in framing is important, as it positions social robots as complementary assistive supports embedded within supportive care practices rather than as incidental distractions or autonomous therapeutic solutions.

Within this perspective, a social robot may be considered a structured clinical tool when its use is linked to an explicit therapeutic objective, implemented through a brief and reproducible protocol within the care pathway, and supervised by healthcare personnel. Its value lies in facilitating engagement and emotional regulation in coordination with clinical routines, rather than functioning as an independent therapeutic agent. Evaluation through relevant outcomes such as anxiety, distress, or cooperation helps situate these interventions within rehabilitation-oriented care while maintaining appropriate safety and privacy safeguards (8).

Evidence from controlled settings further supports this view. In Turkey, a randomized trial with 84 children undergoing ambulatory surgery demonstrated that the presence of an interactive robot reduced anxiety prior to postoperative mobilization and increased parental satisfaction with the care provided (9). From the perspective of clinical practice, this finding is particularly valuable because it links the emotional dimension to functional recovery, a central objective of enhanced recovery protocols following surgery. It is not simply a matter of the child being calmer, but of reducing anxiety in a way that enables more efficient recovery, with direct benefits for hospital stay duration and patient cooperation. In this sense, the clinical relevance of social robots emerges most clearly when emotional support is aligned with concrete care processes and observable engagement.

Even among populations with greater psychological vulnerability, such as children with social anxiety, evidence suggests that interaction with robots fosters prosocial behaviors and openness to contact, positioning these devices as relational mediators with still underexplored potential (10). The robot should not be seen as a substitute for human interaction, but as a facilitator that opens communication channels where anxiety or fear might otherwise close them. These findings are promising, although broader validation across diverse clinical contexts remains necessary.

Moreover, social robotics can be embedded into hospital routines without the need for complex infrastructure or disproportionate investment, functioning as an extension of therapeutic play already recognized in pediatrics (11). Yaren was introduced as an open-source humanoid torso, accessible and anthropometrically close to the proportions of a child. Its design is not a minor technical detail, as it offers a solution that does not depend on proprietary and costly platforms and can be implemented particularly in low- and middle-income countries (12). These examples illustrate why the field is moving toward clinically oriented uses, while also underscoring that feasibility and scalability will shape real-world adoption in pediatric hospitals.

## 3 The voice of caregivers and families

The value of social robots lies not only in the child's direct experience but also in what they represent for those who accompany their process. From the perspective of families, the usefulness of these technologies is measured both in the relief they bring to the child and in the reassurance they provide to parents (13). As noted by (14), the possibility of the robot becoming a “safe space” to express feelings or practice coping techniques gives caregivers the sense of having an additional ally, rather than just a temporary distraction. In this sense, parental acceptance is not a minor detail but a decisive criterion for ensuring that the use of robots can be sustained over time within the hospital environment. This acceptance reflects how families interpret supportive technologies in moments of uncertainty, and it may shape the child's participation, adherence to mobilization or therapy-related tasks, and sustained coping beyond the acute hospital encounter (15).

A similar perspective emerges from healthcare professionals. Hudson et al. (16) show that physicians and nurses recognize the value of robots with adaptive intelligence to accompany invasive procedures but emphasize that these should act as complements, never as replacements. In pediatric settings, this complementarity depends on clear safeguards that protect children and families while ensuring that robots can be integrated without disrupting clinical routines. Robots are more likely to be welcomed when they reinforce, rather than compete with, the caregiver's role by offering relief during critical moments and easing part of the emotional burden carried by families and healthcare teams (17). At the same time, concerns remain about reduced human interaction and data sensitivity in an already delicate care context (18). These issues should not be treated as static barriers, but as practical determinants that shape how social robots can be designed and deployed to respect human boundaries and strengthen the bond between the child, caregivers, and the clinical team.

## 4 Discussion

Social robots should be discussed in pediatric hospitalization not only as an innovation in patient experience, but as a potential contributor to rehabilitation-relevant care. Advances in this area suggest that their most consistent contribution lies in reducing anxiety, stress, and distress, rather than directly relieving pain (19). This finding calls for a reconsideration of the initial hypotheses: robots should not be regarded as “technological analgesics,” but as tools of emotional and pedagogical support that complement medical and psychosocial interventions (10). This distinction is clinically meaningful because improvements in functional outcomes appear to be mediated through emotional regulation, engagement, and increased willingness to participate, rather than through direct therapeutic action. By supporting emotional readiness and reducing avoidance behaviors, these systems may facilitate cooperation, participation in mobilization, and adherence to therapy-related activities, particularly in perioperative and inpatient contexts (9).

An important strength is the high acceptability reported by children and families, who perceive robots as companions, playmates, and mediators of communication. In practice, this acceptability has implications beyond satisfaction: caregiver reassurance and engagement can shape adherence to care routines and sustain coping behaviors throughout hospitalization, influencing the continuity of supportive and rehabilitative care.

At the same time, the evidence base is not yet mature enough to justify one-size-fits-all clinical protocols. Recent reviews point to heterogeneous designs and limited sample sizes, which complicate interpretation and reduce transferability across clinical contexts (20, 21). Technological variability also remains substantial, with a predominance of humanoid robots such as NAO, while more accessible formats have received less systematic evaluation (22). Rather than undermining the field, these limitations clarify what is needed next: reproducible interventions, clearer descriptions of interaction components, and outcomes that reflect both emotional regulation and rehabilitation-oriented engagement.

**Figure 1:**
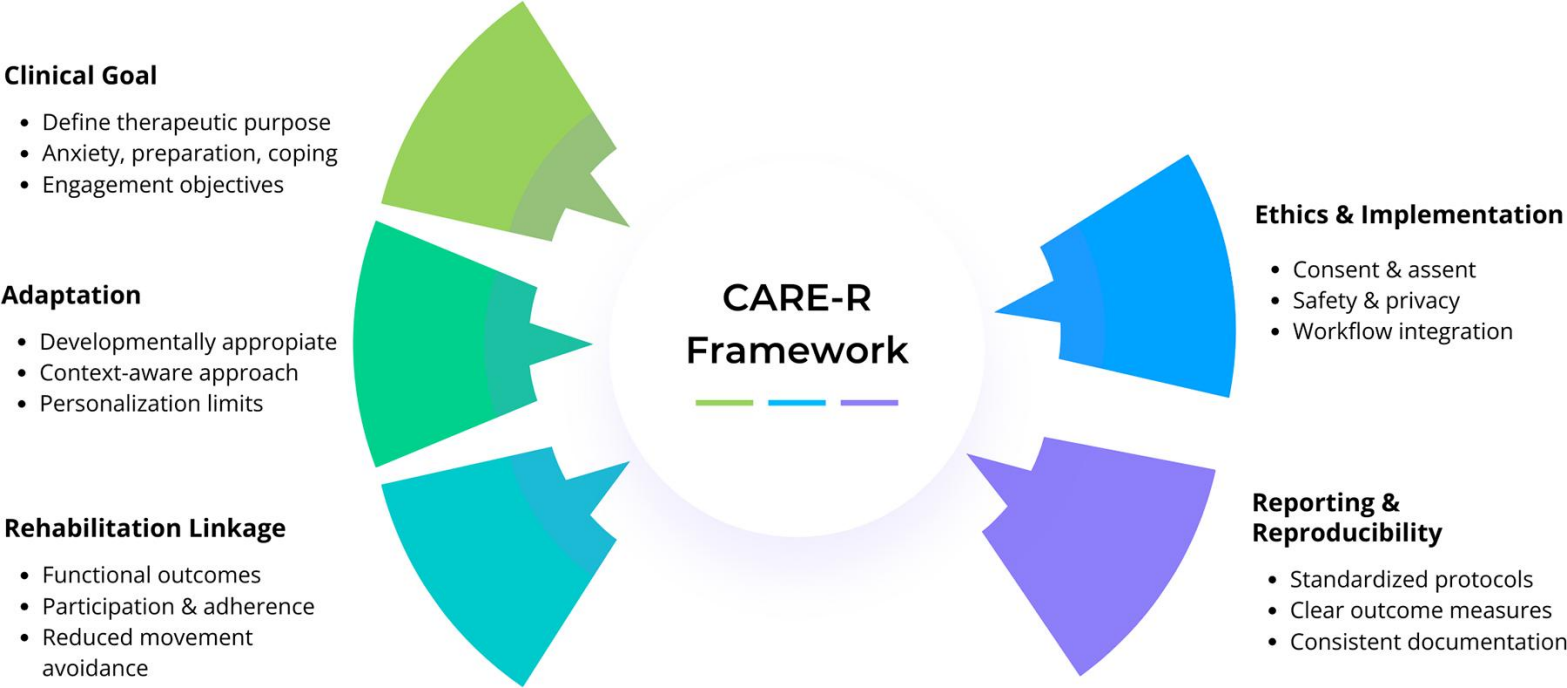
CARE-R framework for clinically meaningful integration of social robots in pediatric hospitalization.

To help bridge the gap between promising findings and practical implementation, we propose the CARE-R framework as a structured approach to guide clinically meaningful use of social robots in pediatric hospitalization (Figure 1). This approach supports decision-making that aligns the intended clinical purpose, context-sensitive interaction design, rehabilitation-relevant outcomes, and implementation safeguards. It also highlights the value of brief, feasible protocols and outcome selection that prioritizes emotional and educational indicators while reserving pain as a secondary endpoint when appropriate (23).

Importantly, sustainable adoption in pediatric hospitals depends on governance conditions, including consent and assent, privacy-by-design, infection control, staff training, and workflow integration (16, 18). This includes clear consent and child assent processes, as well as privacy-by-design practices, particularly when robots interact with children during vulnerable moments of care. Infection control routines, staff training, and workflow integration are equally critical, as they determine whether these systems can be deployed without introducing new risks, increasing workload, or disrupting time-sensitive clinical procedures. Equity must also be considered, since the clinical value of social robots will remain limited if implementation relies on proprietary platforms, specialized personnel, or resources unavailable in public and resource-constrained pediatric units.

Beyond their current limitations, social robots align with the vision of assistive technologies as instruments to “leave no one behind.” When deployed with scientific grounding and equity in mind, they can contribute to a more inclusive form of universal health coverage, where the emotional and educational well-being of hospitalized children is recognized as a legitimate healthcare goal. Thus, the answer seems clear. Yes, social robots can improve the hospital experience of children, provided they are implemented responsibly and supported by evidence.
